# Targeted Wastewater Surveillance during the World Athletics Championship, Oregon, USA, 2022

**DOI:** 10.3201/eid3213.260537

**Published:** 2026-08

**Authors:** Rebecca Falender, Devrim Kaya, Michael Harry, Oumaima Hachimi, Tyler Radniecki, Christine Kelly, Paul R. Cieslak, Andraya Hendrick, David Mickle, Anirudh Bhatia, Brent Kronmiller, Mathew Geniza, Dana Alegre, Justin Elser, Patrick Luedtke, Stacey Jeffries Miles, Nancy Gerloff, Melissa Sutton

**Affiliations:** Oregon Health Authority, Portland, Oregon, USA (R. Falender, P.R. Cieslak, A. Hendrick, M. Geniza, M. Sutton); Oregon State University, Corvallis, Oregon, USA (D. Kaya, M. Harry, O. Hachimi, T. Radniecki, C. Kelly, D. Mickle, A. Bhatia, B. Kronmiller, D. Alegre, J. Elser); Lane County Department of Health and Human Services, Eugene, Oregon, USA (P. Luedtke); Centers for Disease Control and Prevention, Atlanta, Georgia, USA (S. Jeffries Miles, N. Gerloff)

**Keywords:** wastewater surveillance, epidemiologic monitoring, SARS-CoV-2, influenza, hepatitis, viruses, measles virus, Middle East Respiratory Syndrome coronavirus, poliovirus, mass gatherings, World Athletics Championship, Oregon, United States

## Abstract

Targeted wastewater surveillance during the 18th World Athletics Championships in Eugene, Oregon, USA, in 2022 detected influenza A virus, SARS-CoV-2, and hepatitis A virus. Poliovirus detections were inconclusive. Influenza B, hepatitis E, and measles viruses and Middle East respiratory syndrome coronavirus were not detected. Wastewater surveillance augments traditional surveillance to mitigate risks associated with large multinational gatherings.

During July 15–24, 2022, the University of Oregon (Eugene, Oregon, USA) hosted the 18th World Athletics Championships. More than 1,700 athletes from 179 nations competed, and ≈54,000 persons attended the first 3 days of the event (*1*,*2*). During the event, Oregon Health Authority, in collaboration with Oregon State University, Lane County Health and Human Services, University of Oregon, and the city of Eugene, implemented targeted wastewater testing for common or high-consequence pathogens unlikely to be detected through existing surveillance mechanisms: SARS-CoV-2, influenza A and B viruses, hepatitis A and E viruses, measles virus, Middle East respiratory syndrome coronavirus (MERS-CoV), and poliovirus.

## Methods

### Study Design

**Figure 1:**
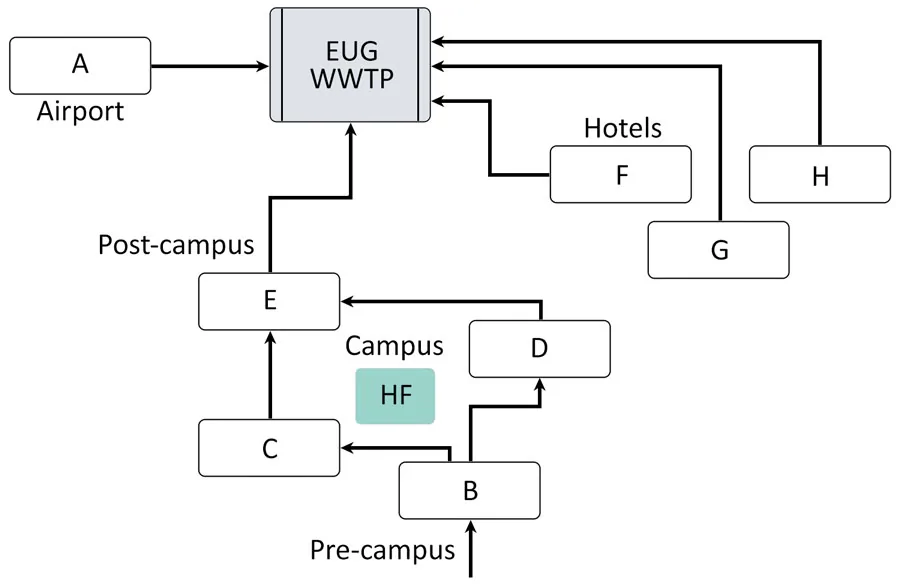
Flow of wastewater and sampling locations during the World Athletics Championship, Eugene, Oregon, USA, 2022. Locations indicate A, Eugene Airport area; B, city of Eugene before water flow enters the University of Oregon campus (precampus); C and D, city of Eugene plus University of Oregon campus (campus); E, city of Eugene, University of Oregon campus, and neighborhood immediately west of the campus (postcampus); F, G, H, hotels and adjacent neighborhood. Location details are available in the Appendix Figure. EUG WWTP, Eugene Wastewater Treatment Plant; HF, Hayward Field.

We adapted Oregon Health Authority and Oregon State University’s existing wastewater surveillance (WS) system for the event. Wastewater samples were collected from Eugene’s wastewater treatment plant (WWTP) influent and 8 microsewershed sites relevant to the event (Figure 1; Appendix Figure). We initiated surveillance ≈2 weeks before the event and continued through 4 weeks after the event (June 28–August 23, 2022). Wastewater samples were collected daily during the event, 2× times/week for 2 weeks before and after the event, and 1× time/week during the final 2 weeks of the surveillance period. We analyzed wastewater data in R Studio version 4.3.1 (https://rstudio.com/products/rstudio). We published results to a public-facing dashboard within 24 hours. We did not make poliovirus results publicly available because confirmatory testing was negative.

### Sample Collection and Analyses

We concentrated 24-hour composite wastewater samples, extracted nucleic acids, and quantified viral concentrations, as previously described (*3*), and conducted droplet digital reverse transcription PCR (ddRT-PCR) (Appendix Tables 1–3). We sequenced samples that contained >4.0 log_10_ gene copies/L of SARS-CoV-2, as previously described (*4*). We analyzed all samples and controls in duplicate, using a limit of detection of 3 droplets for positive samples and controls.

### Poliovirus Cross-Reactivity Analysis

We evaluated in silico cross-reactivity of the poliovirus ddRT-PCRs against 164 reference genomes obtained from the National Center for Biotechnology Information Virus database (https://www.ncbi.nlm.nih.gov/labs/virus), representing 12 enterovirus C types known to have cross-reactivity with other poliovirus assays. We conducted those analyses using Geneious Prime version 2025.2.2 software (https://www.geneious.com) as described previously (*5*,*6*). Potential cross-reactivity required that the forward primer, reverse primer, and probe were located within the same reference sequence with <4 mismatches each, and none occurring in the last 3 bases of the 3′ end.

## Results

**Figure 2:**
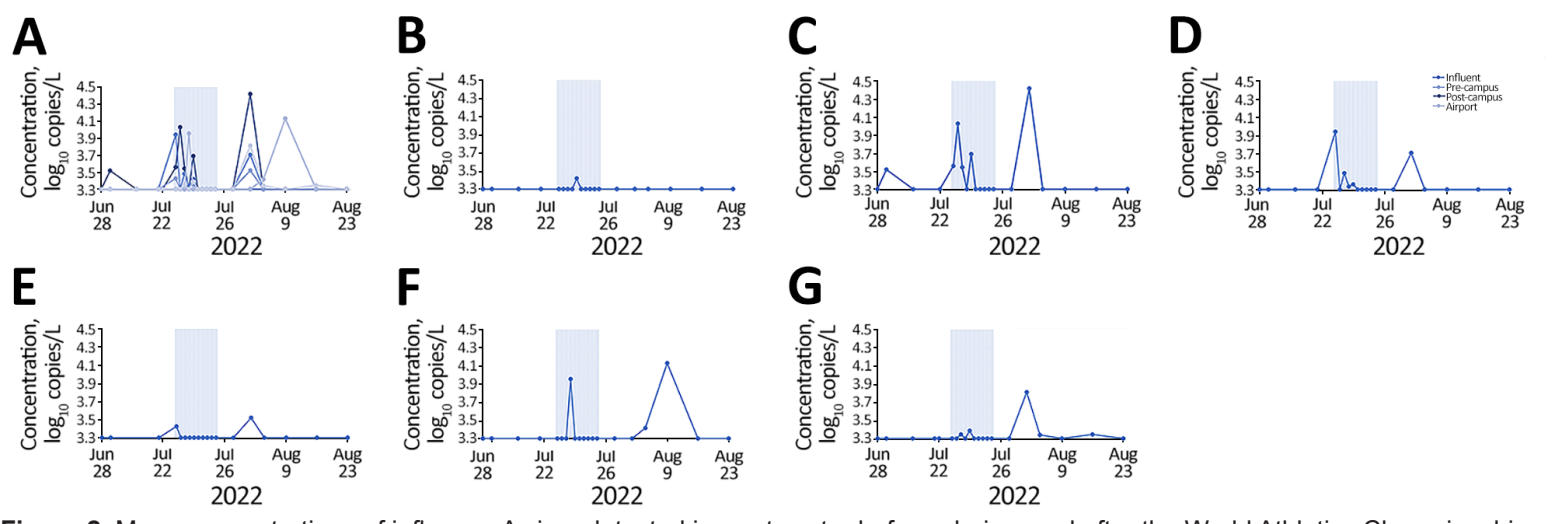
Mean concentrations of influenza A virus detected in wastewater before, during, and after the World Athletics Championship, Eugene, Oregon, USA, 2022. Results are shown by sampling locations: A) all sites; B) Eugene Wastewater Treatment Plant; C) city of Eugene, University of Oregon campus, and neighborhood immediately west of the campus (postcampus); D) city of Eugene plus 2 sites on the University of Oregon campus (on-campus); E) city of Eugene before the point at which water flow enters the University of Oregon campus (precampus); F) Eugene Airport area; G) 3 local hotels and adjacent neighborhood. Location details are available in the Appendix Figure.

**Figure 5:**
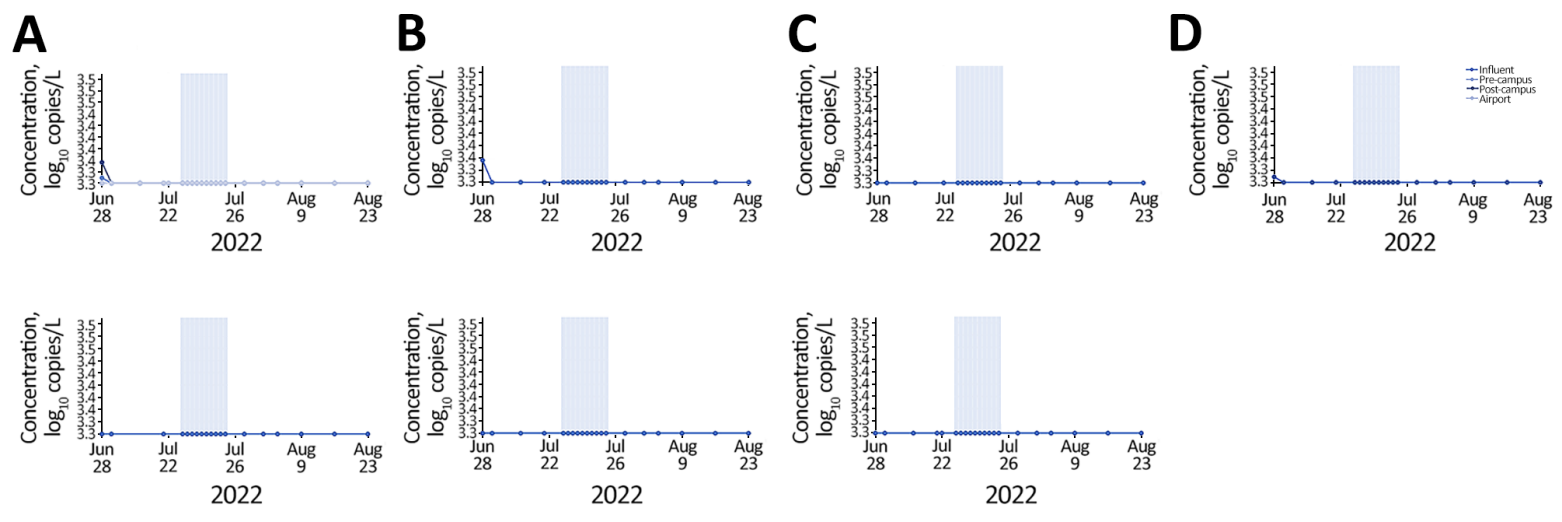
Mean concentrations of hepatitis A detected in wastewater before, during, and after the World Athletics Championship, Eugene, Oregon, USA, 2022. Results are shown by sampling locations: A) all sites; B) Eugene Wastewater Treatment Plant; C) city of Eugene, University of Oregon campus, and neighborhood immediately west of the campus (postcampus); D) city of Eugene plus 2 sites on the University of Oregon campus (on-campus); E) city of Eugene before the point at which water flow enters the University of Oregon campus (precampus); F) Eugene Airport area; G) 3 local hotels and adjacent neighborhood. Location details are available in the Appendix Figure.

**Figure 3:**
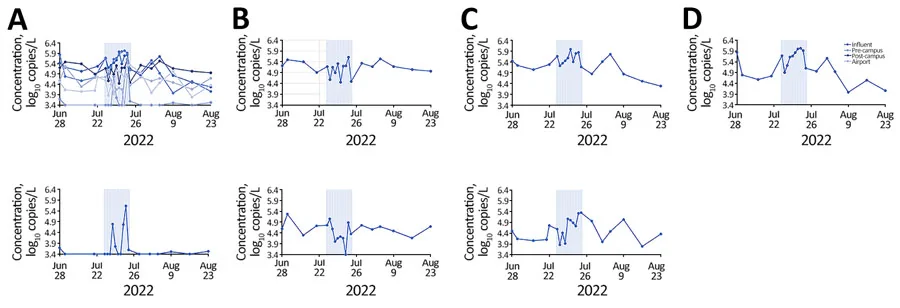
Mean concentrations of SARS-CoV-2 detected in wastewater before, during, and after the World Athletics Championship, Eugene, Oregon, USA, 2022. Results are shown by sampling locations: A) all sites; B) Eugene Wastewater Treatment Plant; C) city of Eugene, University of Oregon campus, and neighborhood immediately west of the campus (postcampus); D) city of Eugene plus 2 sites on the University of Oregon campus (on-campus); E) city of Eugene before the point at which water flow enters the University of Oregon campus (pre-ampus); F) Eugene Airport area; G) 3 local hotels and adjacent neighborhood. Location details are available in the Appendix Figure.

**Figure 6:**
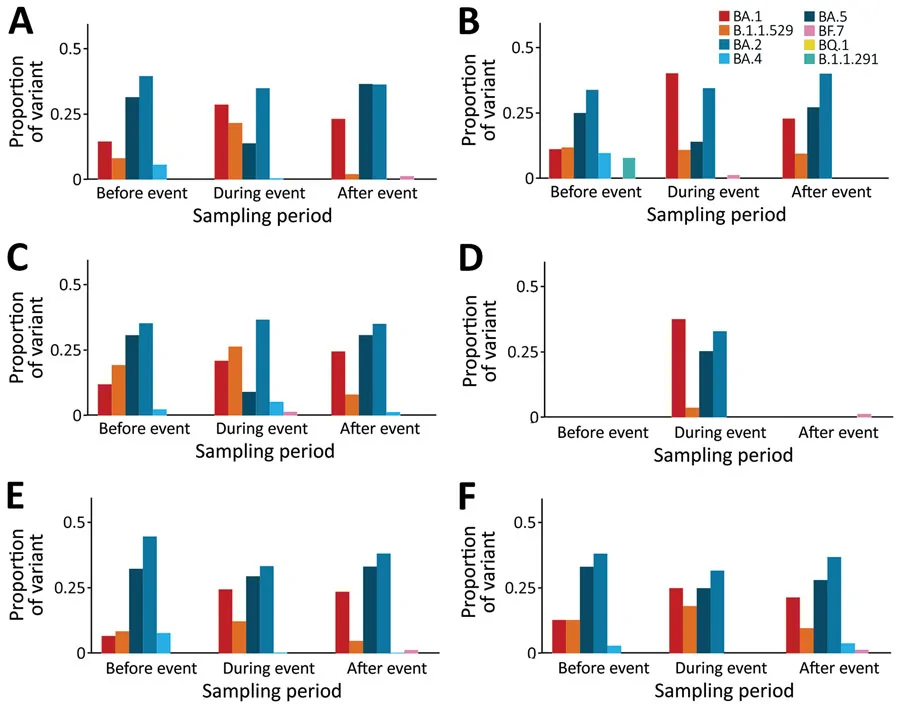
Proportions of SARS-CoV-2 variants detected in wastewater before, during, and after the World Athletics Championship, Eugene, Oregon, USA, 2022. The sampling periods were July 1–14, before the event; July 15–24, during the event; and July 25–August 21, after the event. The variant proportions are aggregated for 3 hotel and 2 on-campus sites. Results are shown by sampling locations: A) Eugene Wastewater Treatment Plant; B) 3 local hotels and adjacent neighborhood; C) Eugene Airport area; D) city of Eugene before the point at which water flow enters the University of Oregon campus (precampus); E) city of Eugene plus 2 sites on the University of Oregon campus (on-campus); F) city of Eugene, University of Oregon campus, and neighborhood immediately west of the campus (postcampus).

**Figure 4:**
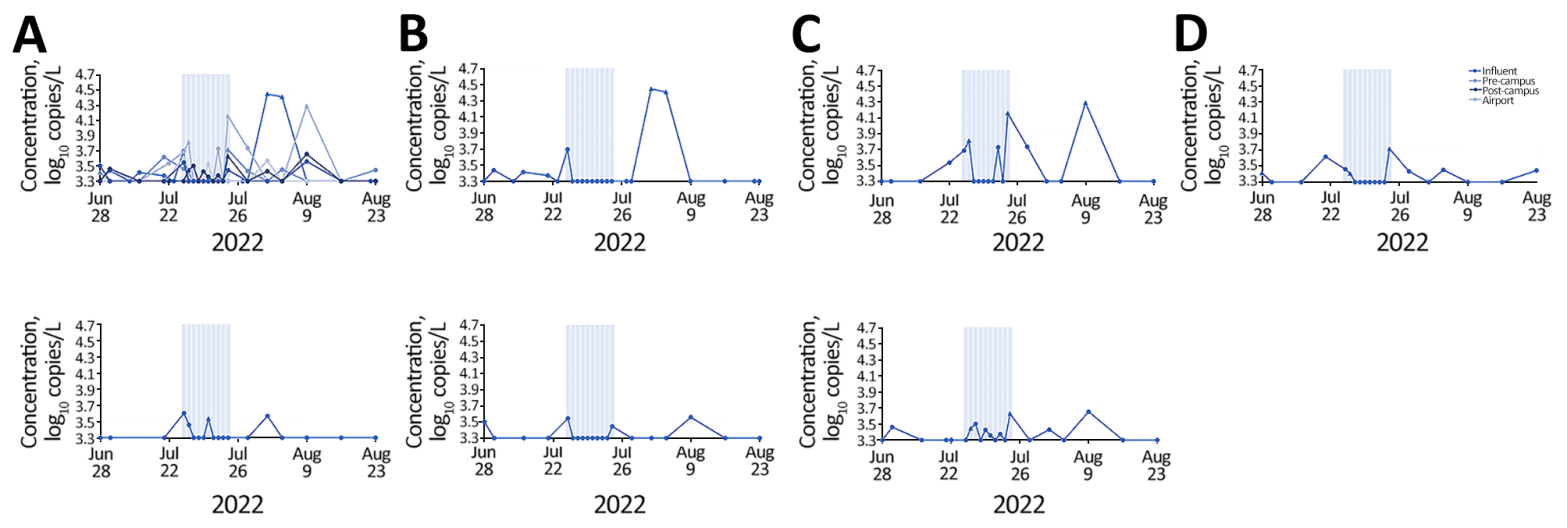
Mean concentrations of poliovirus detected in wastewater before, during, and after the World Athletics Championship, Eugene, Oregon, USA, 2022. Triangles indicate poliovirus samples that tested positive at retest. Poliovirus results were not confirmed by the CDC. Results are shown by sampling locations: A) all sites; B) Eugene Wastewater Treatment Plant; C) city of Eugene, University of Oregon campus, and neighborhood immediately west of the campus (postcampus); D) city of Eugene plus 2 sites on the University of Oregon campus (on-campus); E) city of Eugene before the point at which water flow enters the University of Oregon campus (precampus); F) Eugene Airport area; G) 3 local hotels and adjacent neighborhood. Location details are available in the Appendix Figure.

We collected a total of 178 samples (Table; Figures 2–5). We detected influenza A in 24 (14%) samples (Table; Figure 2): 1 detection before the event, at multiple sites during the event, and intermittently after the event. Despite microsewershed detections, we detected influenza A only once in WWTP influent. We detected SARS-CoV-2 in 147 (83%) samples (Table; Figure 3); sequencing revealed a decrease in the variants BA.2, BA.4, and BA.5 that were most prevalent in Oregon at that time and an increase of previously abundant variants B.1.1.529 and BA.1 (Figure 6). We detected low concentrations of hepatitis A once, 17 days before the start of the event (Table; Figure 5).

**Table:** Summary of pathogen detections and concentrations in wastewater at the World Athletics Championship, Oregon, USA, 2022*

Pathogen	No. detections	No. sites with detections	Average concentration, log_10_ gene copies/L	Range, log_10_ gene copies/L
Influenza A	24	8	3.70	3.38—4.42
SARS-CoV-2	147	9	4.96	3.34—6.92
Poliovirus†	42	9	3.84	3.43—5.04
Hepatitis A	3	2	3.33	3.26—3.37
Hepatitis E	0	0	NA	NA
Influenza B	0	0	NA	NA
Measles	0	0	NA	NA
MERS-CoV	0	0	NA	NA

*MERS-CoV, Middle East respiratory syndrome coronavirus; NA, not applicable. †Results not confirmed by Centers for Disease Control and Prevention.

We detected panpoliovirus at very low concentrations in 42 (24%) samples; however, none were positive on serotype-specific poliovirus assays (Sabin 1, 2, or 3) (Table; Figure 4; Appendix Table 4). For further characterization, we retested 32 samples with higher concentrations of panpoliovirus; 11 of those were positive (Appendix Table 4). We sent those 11 samples for confirmatory testing to the Centers for Disease Control and Prevention (Atlanta, Georgia, USA), where they tested negative on a panpoliovirus quantitative reverse transcription PCR (qRT-PCR). We observed no cross-reactivity between the poliovirus assays and enterovirus C types (Appendix Table 5). Poliovirus concentrations were insufficient for sequencing. We did not detect influenza B, hepatitis E, measles, or MERS-CoV viruses during the surveillance period. We reviewed and posted results on a public-facing dashboard daily and interpreted them in the context of case and syndromic data.

## Discussion

Targeted WS during the WAC documented possible new introductions of influenza A and SARS-CoV-2 variants. Hepatitis A was detected once. Poliovirus detections could not be confirmed. Thirty-three (18%) of 179 participating nations were located entirely in the Southern Hemisphere, where seasonal influenza typically peaks in July and August. After a second cluster of influenza A detections, we found no evidence of further propagation; we detected influenza A once at the WWTP, which suggested that it was not widespread in the community. Trend analysis in influenza percent positivity from 3 clinical laboratories in the region showed no change (*7*). Similarly, we observed no increase in the proportions of emergency department and urgent care visits attributed to influenza-like illness among the 6 facilities within 25 miles of the event (*8*); those results suggested that targeted WS was more sensitive in identifying influenza A when the pathogen was not widespread in the community. 

The WAC reintroduced variants of SARS-CoV-2 that had not been in circulation in Oregon since June 2020 (*9*). Population-level immunity and intervariant competition likely prevented ongoing community transmission (*10*). Most variant proportions returned to preevent baseline proportions after the event.

The detections of panpoliovirus nucleic acids by ddRT-PCR might represent cross-reactivity with nonpolio enteroviruses or true detection of polioviruses at levels too low to be identified by qRT-PCR or serotype-specific assays. WS has documented circulating vaccine-derived poliovirus in several nonendemic countries, including the United States, after importation from countries with ongoing oral poliovirus vaccine use (*11*–*13*). Previous studies have shown ddRT-PCR to have greater analytical sensitivity; limit of detection was significantly lower (in 1 study, more than tenfold lower) than qRT-PCR in detecting wastewater viruses (*14*,*15*). In our study, the poliovirus assays did not cross-react with 12 enterovirus C genomes; it is possible that the assay would cross-react with enteroviruses we did not include in our analysis (Appendix Table 5) (*5*,*6*). No human polio cases were reported in Oregon during the surveillance period.

Because the ddRT-PCR we used was highly sensitive, we inferred that influenza B, hepatitis E, and measles viruses and MERS-CoV were not introduced during the event because they were not detected. No local cases of those diseases were reported to public health during the surveillance period.

This study demonstrated the feasibility and utility of WS for pathogens of public health significance that might not be detected through traditional surveillance systems during a large-scale international event. Through targeted microsewershed surveillance, we detected possible introductions of influenza A and SARS-CoV-2 variants. However, we lacked the metadata to normalize microsewershed viral concentrations by population or flow. Furthermore, because visitors arrived at different times before and during the event, distinctions between event periods are not clear.

Our results strengthen previous studies of WS surrounding large events and provide additional considerations regarding panpoliovirus detections in wastewater (*16*). Our findings regarding increased influenza circulation could specifically indicate mitigation efforts to include alerting healthcare providers to potential influenza outbreaks and ensuring supplies of oseltamivir. Overall, targeted WS should be considered during international events as a tool to enable early outbreak detection and response, inform public health efforts, and mitigate the risk of large gatherings. 

Appendix Additional information about wastewater surveillance during the World Athletics Championship, Oregon, USA, 2022.
