# Supplementary material for: Targeted Wastewater Surveillance during the World Athletics Championship, Oregon, USA, 2022
Source: Emerg Infect Dis. 2026 Aug;32(Suppl 1):S26–31. doi: 10.3201/eid3213.260537 (PMC13585663; doi:10.3201/eid3213.260537)
Supplement: Supplementary file 1 [file 26-0537-Techapp-s1.pdf]

Article DOI: https://doi.org/10.3201/eid3213.260537

EID cannot ensure accessibility for supplementary materials supplied by authors. Readers who have difficulty accessing supplementary content should contact the authors for assistance.

Targeted Wastewater Surveillance during the World Athletics Championship, Oregon, USA, 2022

Appendix

Appendix Table 1. Primers, probes and cycling parameters used for wastewater surveillance during the World Athletics Championship

Target	MG*	Gene target	Oligo name	Oligo type	Sequence (5'-3')†	T_m (°C)	PCR conc. (nM)
SARS-CoV-2‡	1	Nucleocapsid Protein (N1)	nCoV_N1-P	Probe	/FAM-ACCCCGCATTACGTTTGGTGGAC C-BHQ1	60.8	250
			nCoV_N1-F	Forward	GACCCCAAAATCAGCGAAAT	49.7	900
			nCoV_N1-R	Rev	TCTGGTTACTGCCAGTTGAATCTG	55.7	900
SARS-CoV-2†	1	Nucleocapsid Protein (N2)	nCoV_N2-P1	Probe	/5FAM/ACAATTTGC/ZEN/CCCCAGCGCTTCAG/3IABkFQ/	58.8	125
			nCoV_N2-P2	Probe	/5HEX/ACAATTTGC/ZEN/CCCCAGCGCTTCAG/3IABkFQ/	58.8	125
			nCoV_N2-F	Forward	TTACAAACATTGGCCGCAAA	47.7	900
			nCoV_N2-R	Rev	GCGCGACATTCCGAAGAA	50.3	900
RPase†	1	Human RPase Gene (RPase)	RP-P	Probe	/5HEX/TTCTGACCT/ZEN/GAAGGCTCTGCGCG/3IABkFQ/	60.6	250
			RP-F	Forward	AGATTTGGACCTGCGAGCG	53.2	900
			RP-R	Rev	GAGCGGCTGTCTCCACAAGT	55.9	900
Influenza A§	2	Matrix (M1)	InfA-P (fam)	Probe	/FAM/TGCAGTCCT/ZEN/CGCTCACTGGGCACG/3IABkFQ/	67.2	250
			InfA-F1	Forward	CAAGACCAATCYTGTCACCTCTGAC	58.2	450
			InfA-F2	Forward	CAAGACCAATYCTGTCACCTYTGAC	57.6	450
			InfA-R1	Rev	GCATTYTGGACAAAVCGTCTACG	56.9	675
			InfA-R2	Rev	GCATTTTGGATAAAGCGTCTACG	54.5	225
Influenza B‡	2	Non-structural 2 (NS2)	InfB-P1 (fam)	Probe	/FAM/CCAATTCGA/ZEN/GCAGCTGAAACTGCGGTG/3IABkFQ/	63.8	125
			InfB-P2 (hex)	Probe	/HEX/CCAATTCGA/ZEN/GCAGCTGAAACTGCGGTG/3IABkFQ/	63.8	125
			InfB-F	Forward	TCCTCAAYTCACTCTTCGAGCG	57.7	900
			InfB-R	Rev	CGGTGCTCTTGACCAAATTGG	56.8	900
RPase‡	2	Human RPase Gene (RPase)	RP-P	Probe	/5HEX/TTCTGACCT/ZEN/GAAGGCTCTGCGCG/3IABkFQ/	60.6	250
			RP-F	Forward	AGATTTGGACCTGCGAGCG	53.2	900
			RP-R	Rev	GAGCGGCTGTCTCCACAAGT	55.9	900
MERS-CoV (*1*)	3	Nucleocapsid Protein (N)	MCV-N3_Pfam	Probe	/56-FAM/ACCCCTGCG/ZEN/CAAAATG CTGGG/3IABkFQ/	63.3	250
			MCV-N3_F	Forward	GGGTGTACCTCTTAATGCCAATTC	56.6	900
			MCV-N3_R	Rev	TCTGTCCTGTCTCCGCCAAT	58.4	900
Hepatitis E (*2*)	3	HEV ORF 2/3	HEP_E-Pfam	Probe	/56-FAM/TG ATT CTC A/ZEN/G CCC TTC GC/3IABkFQ/	57.2	125
			HEP_E-Phex	Probe	/5HEX/TGATTCTCA/ZEN/GCCCTTCGC/3IABkFQ/	57.2	125
			HEP_E-F	Forward	GGTGGTTTCTGGGGTGAC	55.9	900
			HEP_E-R	Rev	AGGGGTTGGTTGGATGAA	53.8	900

Target	MG*	Gene target	Oligo name	Oligo type	Sequence (5'-3')†	T_m (ºC)	PCR conc. (nM)
Measles (*3*)	4	Virus Nucleocapsid Protein (VN)	Meas_N1_Pfam	Probe¶	/56-FAM/CTGTTAGAG/ZEN/GTTG TCCAGAGTGACCAG/3IABkFQ/	62.0	250
			Meas_N1_F	Forward	CGATGACCCTGACGTTAGCA	54.3	900
			Meas_N1_R	Rev	GCGAAGGTAAGGCCAGATTG	52	900
Hepatitis A (*4*)	4	HEV ORF	HepA_Phex	Probe	/5HEX/CTTAGGCTA/ZEN/ATACTT CTATGAAGAGATGC/3IABkFQ/	55.3	250
			HepA_F	Forward	GGTAGGCTACGGGTGAAAC	55.3	900
			HepA_R	Rev	AACAACTCACCAATATCCGC	53.0	900
Pan poliovirus (*5*,*6*)	4	Capsid Region	PanPV_Pfam	Probe	/56-FAM/TGRTTNAR/ZEN/ideoxyl/G CRTGICCRTTRTT/3IABkFQ	44.6–56.3	125
			PanPV_Phex	Probe	/5HEX/TGRTTNAR/ZEN/ideoxyl/GCRTGICCRTTRTT/3IABkFQ	44.6–56.3	125
			PanPV_F	Forward	TTGGAGTTCTTCACITAITCIMGITTYGAYATG	55.9–60.1	900
Pan poliovirus (*5*,*6*)<br>Vaccine derived polio (*7*)	4<br>5	Capsid Region<br>Sabin 1	PanPV_R	Rev	GGAGCTCCGGGTGGGAYRTACATIATYTGRTAIAC	63.2–68.1	900
			Sabin1_Pfam	Probe	/56-FAM/CGCCCCCAC/ZEN/CGTTTCA CGGA/3IABkFQ	44.6–56.3	125
			Sabin1_F	Forward	AGGTCAGATGCTTGAAAGC	55.9–60.1	900
			Sabin1_R	Rev	CCACTGGCTTCAGTGTTT	63.2–68.1	900
Vaccine derived polio (*7*)	5	Sabin 2	Sabin2_Pfam	Probe	/56-FAM/ATTGGTTCC/ZEN/CCCGACTT CCACCAAT/3IABkFQ	44.6–56.3	125
			Sabin2_Phex	Probe	/5HEX/ATTGGTTCC/ZEN/CCCGACTTCCACCAAT/3IABkFQ	44.6–56.3	125
			Sabin2_F	Forward	CCGTTGAAGGGATTACTAAA	55.9–60.1	900
Vaccine derived polio (*7*)<br>Vaccine derived polio (*7*)	5<br>5	Sabin 2<br>Sabin 3	Sabin2_R	Rev	CGGCTTTGTGTCAGGCA	63.2–68.1	900
			Sabin3_Phex	Probe	/5HEX/TCACTCCCG/ZEN/AAGCAACAG/3IABkFQ	44.6–56.3	125
			Sabin3_F	Forward	AGGGCGCCCTAACTTT	55.9–60.1	900
			Sabin3_R	Rev	TTAGTATCAGGTAAGCTATC	63.2–68.1	900

*MG = Multiplex Group
†I = iosine; M = a or c; Y = c or t; R = a or g; N = a, c, g or t.
‡2019-nCoVCDCddPCRTriplex 350 Probe Assay, Catalog # 12008202 (Bio-Rad Laboratories, https://www.bio-rad.com).
§CDC Influenza SARS-CoV-2 (Flu SC2) Multiplex Assay (CDC, https://www.cdc.gov).
¶The Meas_N1_Pfam probe was modified from the reference sequence by removing the GG at the beginning of the 5' end to increase signal intensity.

Appendix Table 2. One-step thermal cycling conditions for wastewater surveillance during the World Athletics Championship*

Assay	Condition	Reverse transcriptase	Enzyme activation	Denaturation (40 Cycles)	Annealing	Extension	Enzyme inactivation	Droplet stabilization
Hepatitis A and E, influenza A and B, measles, MERS-CoV, poliovirus (Sabin 1,2,3), SARS-CoV-2	Temperature (ºC)	50	95	95	55	55	98	4
	Time	30 min	10 min	30 s	30 s	30 s	10 min	30 min–16 hr
Pan poliovirus	Temperature (ºC)	50	95	95	48	60	98	4
	Time	30 min	5 min	15 s	60 s	15 s	10 min	30 min–16 hr

*Quality Controls: Quality controls included field blanks, filtration blanks, extraction blanks, negative controls (containing only human genomic DNA), positive controls, and no-template controls. A threshold of 10,000 generated droplets was required for sample validity. A limit of detection of three droplets was required for positive samples and controls. All samples and controls were analyzed in duplicate.

Appendix Table 3. Positive controls used for wastewater surveillance during the World Athletics Championship

Target	Positive control	Positive control sequence	Vendor
Hepatitis A	g-block	TATTAACTTGGCTTTCATGAATCTCTTTGATCTTCCACAAGGGGTAGGCTACGGGTGAAACCTCTTAGGCTAATACTTCTAT GAAGAGATGCTTTGGATAGGGTAACAGCGGCGGATATTGGTGAGTTGTTAAGACAAAAACCATTCAA	This study
Hepatitis E	g-block	TAAATTTCCGGCGGTGGTTTCTGGGGTGACCGGGCTGATTCTCAGCCCTTCGCAATCCCCTATATTCATCCAACCAACCC CTTCGCCCCCGATGTCACCGCTGCGGCCGGGGCTGGACCTCGTGTTTTAAT	This study
Influenza A	Inactivated virus*	N/A	Exact Diagnostics, Cat # COVFLU
Influenza B	Inactivated virus*	N/A	Exact Diagnostics, Cat # COVFLU
Measles	g-block	CAATTGATTCAGAGGATCACCGATGACCCTGACGTTAGCATAAGGCTGTTAGAGGTTGTCCAGAGTGACCAGTCACAATC TGGCCTTACCTTCGCATCAAGAGGTACCAACATGGAGGATGAGGCGGACCAATACTTTTC	This study
MERS-CoV	g-block	TCTTACCTTTCCACCTGGGCAGGGTGTACCTCTTAATGCCAATTCCACCCCTGCGCAAAATGCTGGGTATTGGCGGAGAC AGGACAGAAAAATTAATACCGGGAATGGAATTAAGCAACTGGCTCCCAGGTGGTAC	This study
Pan poliovirus	g-block	AAGATACTGTCCAGTTACGGAGGAAATTGGAGTTCTTCACATATTCGAGATTTGACATGGAGTTCACTTTTGTGGTCACCT CAAACTACATTGATGCAAATAACGGACATGCATTGAACCAAGTGTACCAAATTATGTACGTCCCACCCGGAGCTCCAAACT CGGAGCACCTATCCCTGGTAAA	This study
Poliovirus (Sabin 1,2,3)	g-block	ACAGGGGGTTAGGTCAGATGCTTGAAAGCATGATTGACAACACAGTCCGTGAAACGGTGGGGGCGGCAACGTCTAGAGA CGCTCTCCCAAACACTGAAGCCAGTGGACGCCGTTGAAGGGATTACTAAAAATGCATTGGTTCCCCCGACTTCCACCAAT AGCCTGCCTGACACAAAGCCGAGCACAGGGCGCCCTAACTTTGTCACTCCCGAAGCAACAGGATAGCTTACCTGATACTA AGCCAGTGGCCCGGCGCATTCCG	This study
SARS-CoV-2	Inactivated virus*	N/A	Exact Diagnostics, Cat # COVFLU

*Requires extraction before use.

Appendix Table 4. Initial and confirmatory results for wastewater surveillance for pan poliovirus at The World Athletics Championship.

Initial								Re-test				
Date	Site	Sample type	Log copies per L	Upper CI	Lower CI	SE	Log LOD	Log copies per L	Upper CI	Lower CI	SE	Log LOD
8/1/2022	EUG WWTP	Influent	3.96	11.679	-3.76	0.608	3.653	4.919	5.733	4.104	0.0641	3.653
8/4/2022	EUG WWTP	Influent	4.522	4.599	4.445	0.00604	3.653	4.279	5.636	2.923	0.107	3.653
7/24/2022	Hotels	Sewerage	4.079	4.324	3.834	0.0193	3.778	3.981	10.382	-2.421	0.504	3.778
7/24/2022	Post-campus	Sewerage	4.341	8.297	0.384	0.311	3.556	3.955	12.845	-4.935	0.7	3.556
7/24/2022	Campus	Sewerage	4.295	4.55	4.039	0.0201	3.556	3.95	12.779	-4.879	0.695	3.556
7/16/2022	Post-campus	Sewerage	3.758	10.145	-2.629	0.503	3.556	3.844	11.323	-3.635	0.589	3.556
7/16/2022	Hotels	Sewerage	3.771	7.51	0.033	0.294	3.778	3.681	6.275	1.088	0.204	3.778
7/20/2022	Pre-campus	Sewerage	3.432	5.682	1.183	0.177	3.556	3.621	8.262	-1.02	0.365	3.556
8/9/2022	Post-campus	Sewerage	5.044	9.859	0.229	0.379	3.653	3.515	5.587	1.443	0.163	3.653
7/16/2022	Campus	Sewerage	3.544	7.209	-0.122	0.288	3.556	3.459	6.052	0.867	0.204	3.556
6/28/2022	Campus	Sewerage	3.659	9.799	-2.48	0.483	3.477	3.374	5.889	0.859	0.198	3.477
7/22/2022	Post-campus	Sewerage	3.959	10.087	-2.169	0.482	3.778	Negative				3.778
7/17/2022	Hotels	Sewerage	3.725	6.87	0.579	0.248	3.778	Negative				3.778
7/28/2022	Post-campus	Sewerage	4.099	13.591	-5.392	0.747	3.653	Negative				3.653
7/24/2022	Hotels	Sewerage	3.722	8.418	-0.975	0.37	3.653	Negative				3.653
6/30/2022	EUG-Inf	Influent	3.598	7.956	-0.76	0.343	3.556	Negative				3.556
7/6/2022	EUG-Inf	Influent	3.777	10.403	-2.849	0.521	3.556	Negative				3.556
7/15/2022	Post-campus	Sewerage	4.099	14.817	-6.619	0.844	3.556	Negative				3.556
7/11/2022	Campus	Sewerage	3.821	4.781	2.861	0.0755	3.556	Negative				3.556
8/4/2022	Campus	Sewerage	3.932	12.532	-4.668	0.677	3.556	Negative				3.556
8/23/2022	Campus	Sewerage	3.907	12.185	-4.371	0.652	3.556	Negative				3.556
7/15/2022	Pre-campus	Sewerage	3.933	12.543	-4.677	0.678	3.556	Negative				3.556
7/16/2022	Pre-campus	Sewerage	3.641	8.537	-1.256	0.385	3.556	Negative				3.556
7/11/2022	Campus	Sewerage	4.094	4.762	3.425	0.0526	3.556	Negative				3.556
7/15/2022	Campus	Sewerage	3.966	6.325	1.607	0.186	3.556	Negative				3.556
7/15/2022	Airport	Sewerage	4.093				3.556	Negative				3.556
7/24/2022	Airport	Sewerage	3.614	8.17	-0.942	0.359	3.556	Negative				3.556
8/9/2022	Airport	Sewerage	3.843	11.314	-3.628	0.588	3.556	Negative				3.556
7/20/2022	Hotels	Sewerage	3.675	3.972	3.379	0.0233	3.556	Negative				3.556
7/17/2022	Hotels	Sewerage	3.91	12.234	-4.414	0.655	3.556	Negative				3.556
7/22/2022	Hotels	Sewerage	3.765	10.245	-2.714	0.51	3.556	Negative				3.556
8/9/2022	Hotels	Sewerage	3.673	8.975	-1.63	0.417	3.556	Negative				3.556
7/11/2022	EUG-Inf	Influent	3.432	5.674	1.189	0.176	3.556					
7/15/2022	EUG-Inf	Influent	3.685	9.139	-1.77	0.429	3.556					
7/12/2022	Post-campus	Sewerage	3.526	5.732	1.32	0.174	3.653					
7/28/2022	Campus	Sewerage	3.556	7.376	-0.264	0.301	3.556					
8/1/2022	Pre-campus	Sewerage	3.558	6.174	0.942	0.206	3.653					
6/28/2022	Airport	Sewerage	3.492	7.504	-0.52	0.316	3.477					
6/30/2022	Hotels	Sewerage	3.774	5.928	1.62	0.17	3.556					
7/19/2022	Hotels	Sewerage	3.666	6.061	1.271	0.188	3.778					
8/9/2022	Hotels	Sewerage	4.18	4.949	3.411	0.0605	3.653					
8/1/2022	Hotels	Sewerage	3.678	6.224	1.131	0.2	3.778					

Appendix Table 5. Results from in silico cross-reactivity analysis of poliovirus assays using reference genomes from twelve enterovirus C types.

		Pan-poliovirus				Sabin 1				Sabin 2				Sabin 3			
Enterovirus C type	Reference genomes	Probe	Forward primer	Rev primer	Amp*	Probe	Forward primer	Rev primer	Amp*	Probe	Forward primer	Rev primer	Amp*	Probe	Forward primer	Rev primer	Amp*
Coxsackie A1	PP756358, PP756359, PP756360, PP756361, PP756362, PP756363, PP756364, MK250423, JX174176, JX174177, AF499635	0	0	2	NO	0	0	0	NO	0	0	0	NO	0	0	0	No
Coxsackie A13	JF260920, JF260921, JF260922, JF260923, DQ995635, DQ995636, DQ995637, DQ995638, DQ995639, DQ995640, DQ995641, DQ995642, DQ995643, DQ995644, AF465511, AF499637	0	0	4	NO	0	0	0	NO	0	0	0	NO	0	0	0	NO
Coxsackie A19	PP756354, PP756355, PP756356, PP756357, MT175706, MF678294, AF499641, AB828290	0	0	3	NO	0	0	0	NO	0	0	0	NO	0	0	0	NO
Coxsackie A22	PP756348, PP756349, PP756350, PP756351, PP756352, PP756353, OM963010, OM963011, JN542510, DQ995647, DQ995648, AF499643	0	0	5	NO	0	0	0	NO	0	0	0	NO	0	0	0	NO
Coxsackie A24	PP548237, PP548238, PP548239, PP548240, PP548242, PP028461,	0	0	13	NO	0	0	0	NO	0	0	0	NO	0	0	0	NO

Enterovirus C type	Reference genomes	Pan-poliovirus Probe	Pan-poliovirus Forward primer	Pan-poliovirus Rev primer	Pan-poliovirus Amp*	Sabin 1 Probe	Sabin 1 Forward primer	Sabin 1 Rev primer	Sabin 1 Amp*	Sabin 2 Probe	Sabin 2 Forward primer	Sabin 2 Rev primer	Sabin 2 Amp*	Sabin 3 Probe	Sabin 3 Forward primer	Sabin 3 Rev primer	Sabin 3 Amp*
	OR803779, OR803780, OR633288, OR361387, OR361388, OR361389, OR361390, MZ171074, MZ171075, MZ171076, MZ171077, MZ171078, MZ171079, MZ171080, MZ171081, MZ171082, MZ171083, MZ171084, MZ171085, MZ171086, MZ171087, MZ171088, MZ171089, MZ171090, MZ171091, MZ171092, MZ171093, MZ171094, MZ171095, MZ171096, MG880745, MG880747, MG880748, MG880749, MG880750, MG880751, MG880752, MF419263, KR399988, KR478685, KU183495, KF667358, KF667359, KF667360, KF667361, KF725085, AB769152, AB769154, AB769156, AB769159, AB769160, AB769161, AB769162, AB769163,																

		Pan-poliovirus				Sabin 1				Sabin 2				Sabin 3			
Enterovirus C type	Reference genomes	Probe	Forward primer	Rev primer	Amp*	Probe	Forward primer	Rev primer	Amp*	Probe	Forward primer	Rev primer	Amp*	Probe	Forward primer	Rev primer	Amp*
	AB769164, AB769165, JF742576, JF742577, JF742578, JF742579, JN228097, EF026081, DQ443001, DQ443002, AY876912, AY876913, D90457																
Enterovirus C104	PP756374, PP756375, PP756376, PP756377, PP756378, PP756379, MZ092702, MZ092704, KR815824, JX982253, JX982254, JX982255, JX982256, JX982257, JX982258, JX982259, AB686524	0	0	0	NO	0	0	0	NO	0	0	0	NO	0	0	0	NO
Enterovirus C105	JX393302, PV604347, PP756367, PP756368, MH229997	0	0	0	NO	0	0	0	NO	0	0	0	NO	0	0	0	NO
Enterovirus C109	PP756366, MH128992, MH128993, GQ865517, JN900470	0	0	0	NO	0	0	2	NO	0	0	0	NO	0	0	0	NO
Enterovirus C113	KC344833, KC344834	0	0	0	NO	0	0	1	NO	0	0	0	NO	0	0	0	NO
Enterovirus C116	PQ119791, ON809572, JX514942	0	0	0	NO	0	0	0	NO	0	0	0	NO	0	0	0	NO
Enterovirus C117	PP756369, PP756370, PP756371, PP756372, PP756373, MT338567, MK089787, JX262382	0	0	0	NO	0	0	0	NO	0	0	0	NO	0	0	0	NO

Enterovirus C type	Reference genomes	Pan-poliovirus				Sabin 1				Sabin 2				Sabin 3			
		Probe	Forward primer	Rev primer	Amp*	Probe	Forward primer	Rev primer	Amp*	Probe	Forward primer	Rev primer	Amp*	Probe	Forward primer	Rev primer	Amp*
Enterovirus C118	JX678288, JX961708, JX961709, JX393301	0	0	0	NO	0	0	0	NO	0	0	0	NO	0	0	0	NO

*In silico genome alignment on forward, reverse, and probe binding sites with the potential for amplification

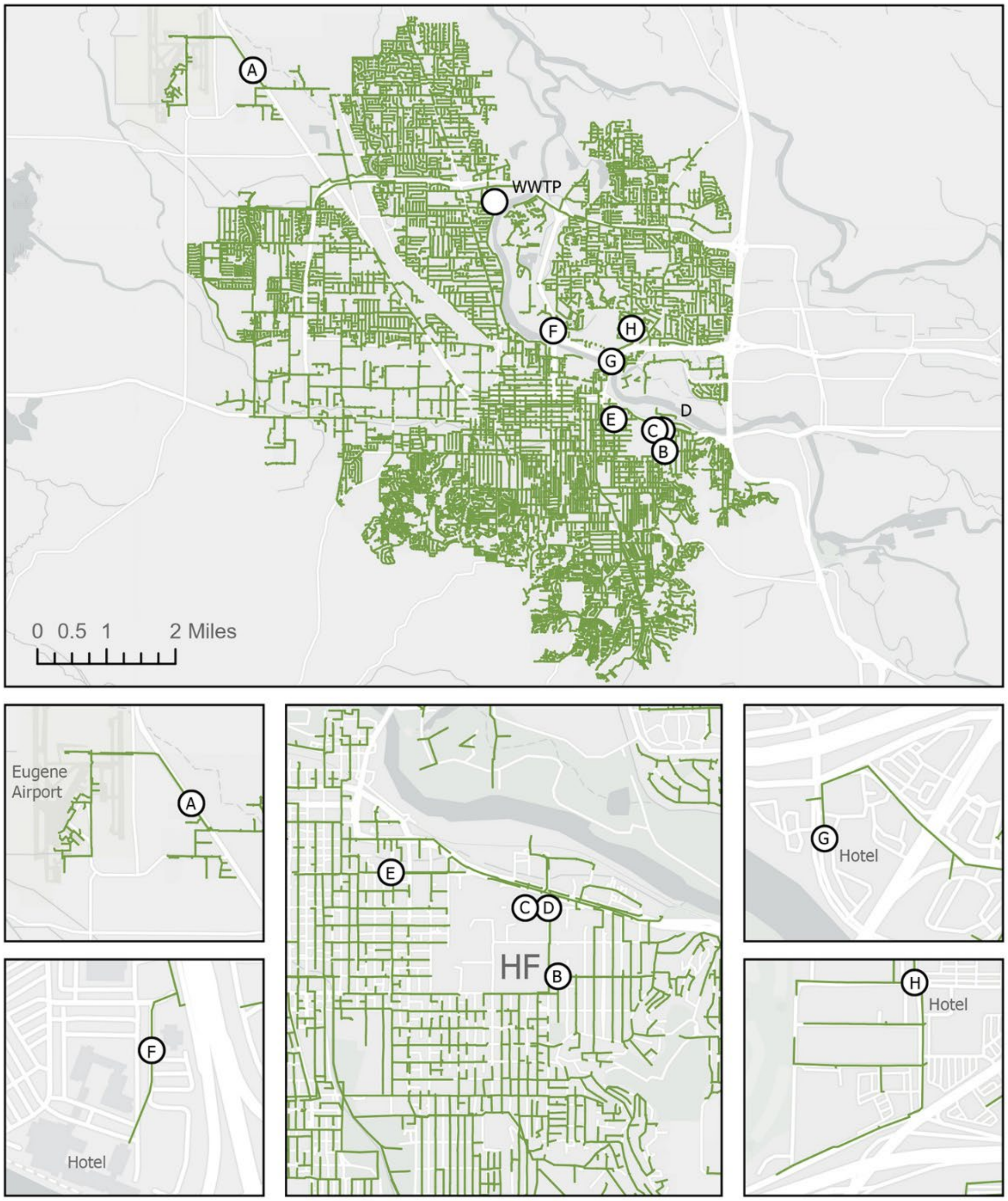

Appendix Figure. Wastewater sampling locations during the World Athletics Championship, sewer lines, and description of catchment area. Location A includes wastewater from the airport and airport administrative buildings only: Highway 99 north of East Enid Rd and south of Awbrey Ln. Location B includes wastewater from the Eugene community prior to entering the University of Oregon campus (pre-campus): Columbia Alley and East 17th Ave. Location C includes wastewater from Pre-campus (B) as well as campus wastewater near Deschutes Hall. No additional city wastewater inputs (campus): Deschutes Hall on 13th Ave. Location D includes wastewater from Pre-campus (B) as well as campus wastewater near and including Hayward Field (HF). No additional city wastewater inputs (campus): Agate St and 13th Ave. Location E includes wastewater from pre-campus (B), campus (C), campus (D) as well as city input

from the neighborhood immediately to the west of the University of Oregon campus (post-campus): 11th Ave and Paterson St. Location F isolates wastewater from a single hotel (hotel region): Valley River Way south of Valley River Dr. Location G isolates wastewater from a single hotel (hotel region): Club Rd north of Coburg Rd. H includes wastewater from hotel and adjacent neighborhood (hotel region): Oakway Rd and Fairway Loop. Location WWTP includes all wastewater from the cities of Eugene and Springfield, Oregon. WWTP, Eugene Wastewater Treatment Plant. Maps produced in ArcGIS Pro version 3.2.2. (Esri Inc., https://www.esri.com). Basemap source: Esri, "Gray Canvas" [basemap], accessed 2026 May 12.

References

1. Lu X, Whitaker B, Sakthivel SK, Kamili S, Rose LE, Lowe L, et al. Real-time reverse transcription-PCR assay panel for Middle East respiratory syndrome coronavirus. J Clin Microbiol. 2014;52:67–75. PubMed https://doi.org/10.1128/JCM.02533-13

2. Germer JJ, Ankoudinova I, Belousov YS, Mahoney W, Dong C, Meng J, et al. Hepatitis E virus (HEV) detection and quantification by a real-time reverse transcription-PCR assay calibrated to the World Health Organization standard for HEV RNA. J Clin Microbiol. 2017;55:1478–87. PubMed https://doi.org/10.1128/JCM.02334-16

3. Benschop KSM, van der Avoort HG, Jusic E, Vennema H, van Binnendijk R, Duizer E. Polio and measles down the drain: environmental enterovirus surveillance in the Netherlands, 2005 to 2015. Appl Environ Microbiol. 2017;83:e00558-17. PubMed https://doi.org/10.1128/AEM.00558-17

4. Jothikumar N, Cromeans TL, Sobsey MD, Robertson BH. Development and evaluation of a broadly reactive TaqMan assay for rapid detection of hepatitis A virus. Appl Environ Microbiol. 2005;71:3359–63. PubMed https://doi.org/10.1128/AEM.71.6.3359-3363.2005

5. Sun H, Harrington C, Gerloff N, Mandelbaum M, Jeffries-Miles S, Apostol LNG, et al. Validation of a redesigned pan-poliovirus assay and real-time PCR platforms for the global poliovirus laboratory network. PLoS One. 2021;16:e0255795. PubMed https://doi.org/10.1371/journal.pone.0255795

6. Kilpatrick DR, Yang CF, Ching K, Vincent A, Iber J, Campagnoli R, et al. Rapid group-, serotype-, and vaccine strain-specific identification of poliovirus isolates by real-time reverse transcription-PCR using degenerate primers and probes containing deoxyinosine residues. J Clin Microbiol. 2009;47:1939–41. PubMed https://doi.org/10.1128/JCM.00702-09

7. Gerloff N, Sun H, Mandelbaum M, Maher C, Nix WA, Zaidi S, et al. Diagnostic assay development for poliovirus eradication. J Clin Microbiol. 2018;56:e01624-17. PubMed https://doi.org/10.1128/JCM.01624-17
